# Case report: Novel homozygous HPGD variant leads to primary hypertrophic osteoarthropathy with intussusception and acro-osteolysis in a Chinese family

**DOI:** 10.3389/fped.2023.1063244

**Published:** 2023-03-09

**Authors:** Yi Liu, Yinghui Wu, Hongxia Tang, Fan Liu, Yali Wu, Shasha Wang, Yan Ding, Wei Yin

**Affiliations:** ^1^ Department of Rheumatology and Immunology, Wuhan Children's Hospital (Wuhan Maternal and Child Healthcare Hospital), Tongji Medical College, Huazhong University of Science & Technology, Huazhong, China; ^2^ Department of Anesthesiology, Wuhan Children's Hospital (Wuhan Maternal and Child Healthcare Hospital), Tongji Medical College, Huazhong University of Science & Technology, Huazhong, China

**Keywords:** acro-Osteolysis, HPGD, intussusception, primary hypertrophic osteoarthropathy (PHO), vitamin d deficiency (VDD)

## Abstract

**Objective:**

To perform molecular genetic analysis of a patient diagnosed with primary hypertrophic osteoarthropathy (PHO) with malnourishment, intussusception, and acro-osteolysis.

**Case presentation:**

At the age of 7 years, a boy born to a consanguineous couple was diagnosed with PHO attributed to delayed closure of the cranial suture, eczema, clubbing of fingers, and swelling of the knee and ankle. Clinical characteristics and follow-up data for 3 years were collected and analyzed. Trio whole-exome sequencing (WES) and copy number variant sequencing were used to screen for causative genetic variants. Candidate variants of the patient and his parents were confirmed by Sanger sequencing. When he was 7 years old, trio WES found that he had biallelic novel variants c.498 + 1G > A, inherited from his parents, in the *HPGD* gene. The patient was markedly malnourished. Ultrasonography and computed tomography showed intussusception with a gradual expansion of the duodenum, localized intestinal wall thickening, and acro-osteolysis. Cross-sectional blood tests showed that the patient had continuously decreased levels of serum 25-hydroxy vitamin D and serum ferritin at the age of 7and 10 years.

**Conclusion:**

PHO due to HPGD defects is rare in pediatric patients, and finding homozygous novel c.498 + 1G > A has expanded the spectrum of causative variants of HPGD and provided a clue for genotype-phenotype correlation analysis. Similar to mouse model results, human HPGD deficiency may also cause abnormal digestive tract development, and related secondary vitamin D deficiency and acro-osteolysis should be considered in HPGD-related PHO.

## 1. Introduction

Primary hypertrophic osteoarthropathy (PHO) is a disease characterized by clubbing of fingers and toes, long periosteal hyperplasia, and skin thickening, with an estimated prevalence of 0.16%. PHO is believed to be related to biallelic pathogenic variants in *HPGD* and *SLCO2A1* (1, 2). The former encodes 15-hydroxyprostaglandin dehydrogenase, a key enzyme for prostaglandin metabolism, whereas the latter encodes a prostaglandin transporter that regulates the distribution and level of prostaglandin in the body. Biallelic *HPGD* mutations can cause prostaglandin metabolic disorders, thereby causing prostaglandins, especially prostaglandin E2 (PGE2), to accumulate excessively in patients. Excessive prostaglandins inhibit osteoblast apoptosis in bone tissue, promote keratinocyte proliferation, pachydermia, and connective tissue hyperplasia in skin tissue, and participate in inflammatory reactions, leading to corresponding clinical symptoms (3, 4).

This study identified a homozygous novel *HPGD* c.498 + 1(IVS5)G > A in a patient diagnosed with PHO with gastrointestinal abnormalities and acro-osteolysis, and is the first report of this mutation among cases of *HPGD* defects.

## 2. Case description

### 2.1. Patient

Case data were obtained from a child with PHO who visited the Department of Rheumatology and Immunology of Wuhan Children's Hospital.

The study was approved by the Ethics Committee of Wuhan Children's Hospital, affiliated with Huazhong University of Science and Technology (2022R053).

### 2.2. Case presentation

The proband was a male child with congenital clubbing of fingers and toes, born to a consanguineous couple. The patient had eczema during infancy, and closure of the posterior cranial suture was delayed until the age of 3 years. At age of 5 years, he had abnormal gait during toe walking, symmetrical swelling, and pain in the knee joints, and he could not squat. The patient was diagnosed with juvenile idiopathic arthritis at a local hospital. He was treated with non-steroidal anti-inflammatory drugs and methotrexate for 2 years; however, his condition did not improve.

**Figure 1:**
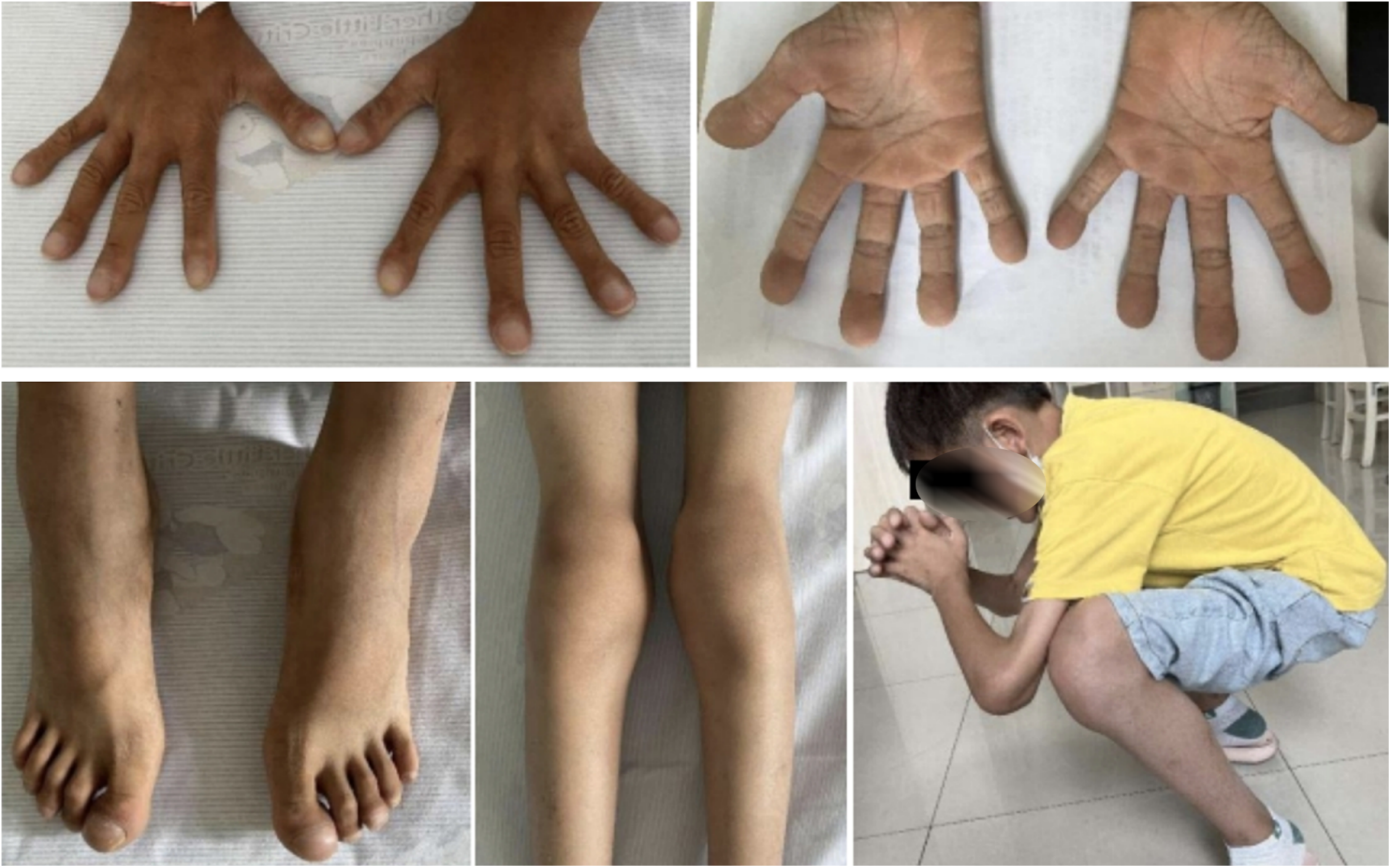
Photographs show clubbed fingers and toes, thickened palm skin, swollen knee joints, and limited flexion in the patient.

**Figure 2:**
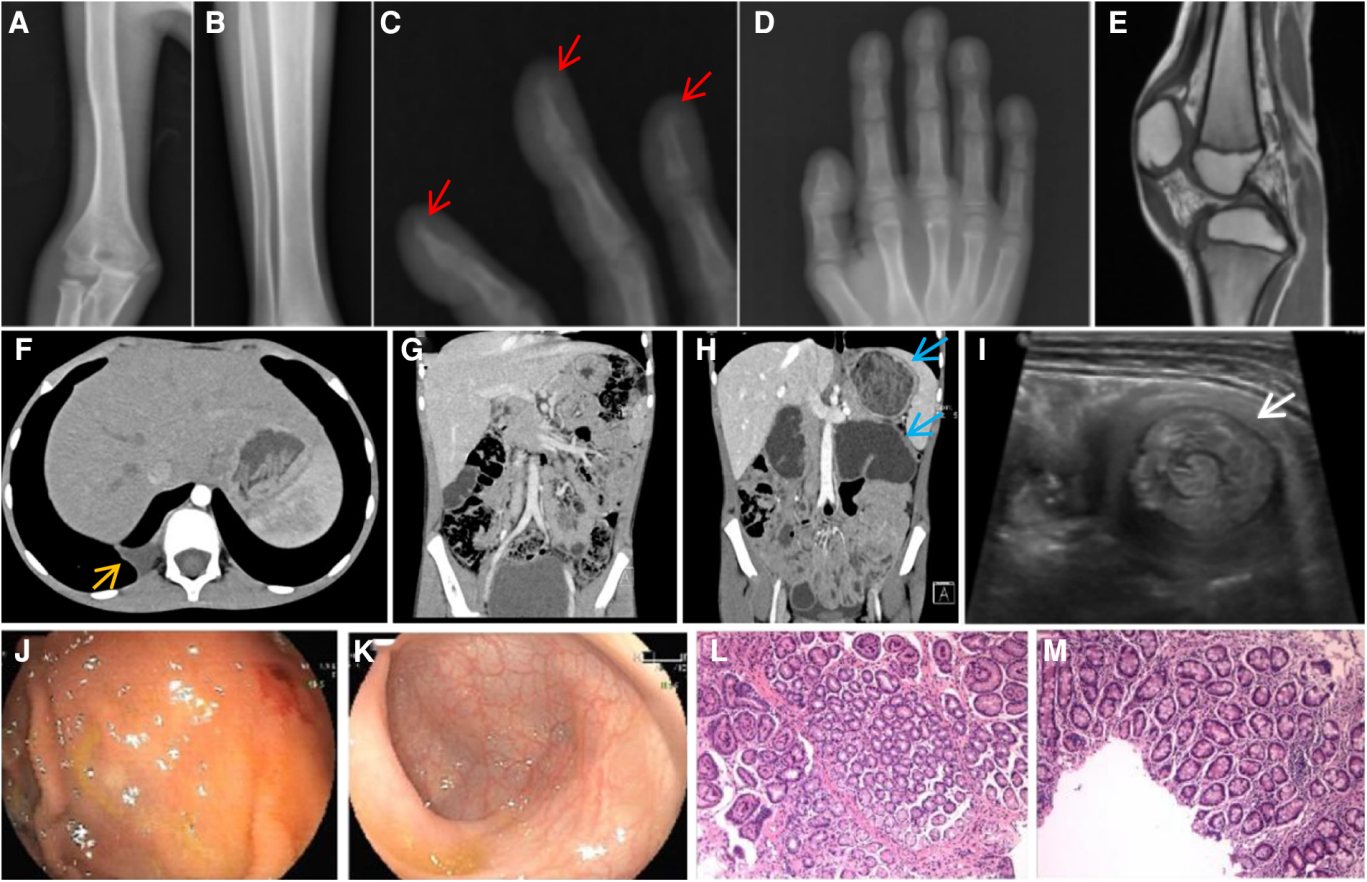
The long bone fragments of the child's hands, feet, and limbs show thickening of the periosteum, osteoporosis, and acro-osteolysis of the phalanges (arrow), swelling knee joint, and enlarged patellar bone with surrounding tissue hyperplasia (**A–E**). Lung computed tomography indicates local pleural thickening and traction (**F**). Compared with the results in 2019, food retention and duodenal dilation were remarkable by imaging in 2022 (**G,H**). Abdominal color ultrasound shows that the jejunum is lengthy, the local intestinal wall is thickened, and it gathers into a mass. There is a transcendental intussusception, which can be unzipped by itself (**I**). Electronic gastroenteroscopy revealed a rough gastric mucosa (**J**) and smooth colonic mucosa (**K**). Pathological biopsy of gastric and intestinal mucosal tissues showed mild changes in chronic inflammation (**L,M**).

In 2019, at 7 years old, the patient was referred to our hospital for further diagnosis. At that time, he still experienced joint swelling, pain, and abnormal walking posture. He did not have any other discomfort, including respiratory symptoms such as coughing and gastrointestinal symptoms such as vomiting, abdominal pain, and diarrhea. Physical examination revealed clubbed fingers and toes with rough bilateral skin and swollen, soft, and movement-restricted knee joints. He had mildly thickened skin but no eczema, acne, or seborrheic dermatitis (Figure 1). No abnormalities were identified in the lungs and abdomen during the physical examination. Imaging examination revealed a significantly thickened periosteum in the long bone, knee joint swelling with an enlarged patellar bone, and surrounding tissue hyperplasia (Figures 2A-E). Lung computed tomography (CT) indicated local pleural thickening and traction (Figure 2F). Intussusception was detected by abdominal CT (Figure 2G). Laboratory tests revealed decreased hemoglobin, ferritin, and active vitamin D levels and a mildly increased erythrocyte sedimentation rate (Table 1).

**Table 1:** Comparison of clinical and laboratory results from 2019 to 2022.

	2019	2022	Reference
Height (cm)	128 (P_50_)^a^	141 (P_50_)^a^	
Weight (kg)	**22** (P_35_)^a^	**25** (<P_5_)^a^	
WBC (×10^9^/L)	7.54	**3.75**	4.3–11.3
Hb (g/L)	114	113	118–156
CRP (mg/L)	2.65	**4.67**	0–3
ESR (mm/h)	**18**	8	0–15
IL-6 (pg/ml)	4.87	**27.54**	0–20.9
TNF-а (pg/ml)	2.64	0.65	0–5.5
Ferritin (ng/ml)	**13.09**	**16.3**	27–375
ALP (U/L)	467	241	146–500
Ca (mmol/L)	2.33	2.05	2–3
*P* (mmol/L)	1.52	1.66	1–1.9
SCr (*μ*mol/L)	34.3	35.5	27–62
PTH (pg/mL)	13.6	15.7	14–72
25-OH-Vit D (ng/ml)	**25.84**	**29.15**	>30
ALT (U/L)	21	17	9–60
TG (mmol/L)	**2.48**	1.37	0.32–1.46

^a^ The height of the child was at the normal average level in 2019 and 2022; at the age of 7 years, the weight was in the 35th percentile (19–32 kg, P_50_ was 24 kg), and at the age of 10 years, the weight was less than the 5th percentile (24–50 kg, P_50_ was 34 kg).

WBC, white blood cell; Hb, hemoglobin; CRP, C-reactive protein; ESR, erythrocyte sedimentation rate; IL-6, interleukin-6; TNF-a, tumor necrosis factor-alpha; ALP, alkaline phosphatase; SCr, serum creatinine; PTH, parathyroid hormone; 25-OH-Vit D, 25-hydroxy vitamin D, ALT, alanine transaminase.

The bold font indicates outliers.

#### 2.2.1. Genetic analysis

Peripheral venous blood was collected from the patient and his parents and sent to the Chigene Translational Medicine Research Center (Chigene Ltd., Beijing, China) for genetic testing. For whole-exome sequencing (WES), xGen® Exome Research Panel v1.0 (IDT, Iowa, USA) was used for exomic library construction; for copy number variant sequencing (CNV-seq), genomic DNA was sheared, the fragment end was repaired, A-tailing was added, and then amplified to construct a genome-wide library. Next-generation sequencing was performed on NovaSeq6000 (Illumina, San Diego, CA, USA), and data processing, quality control, single nuclear variants (SNVs) and indels shorter than or equal to 50 bp calling were performed following the manufacturer's instructions and previously published methods. All variants were updated to the Chigene Inheritance Disease Analysis Cloud Platform (https://cloud.chigene.org/) and interpreted and ranked according to the American College of Medical Genetics (ACMG) clinical practice guidelines and correlated phenotypes, respectively.

**Figure 3:**
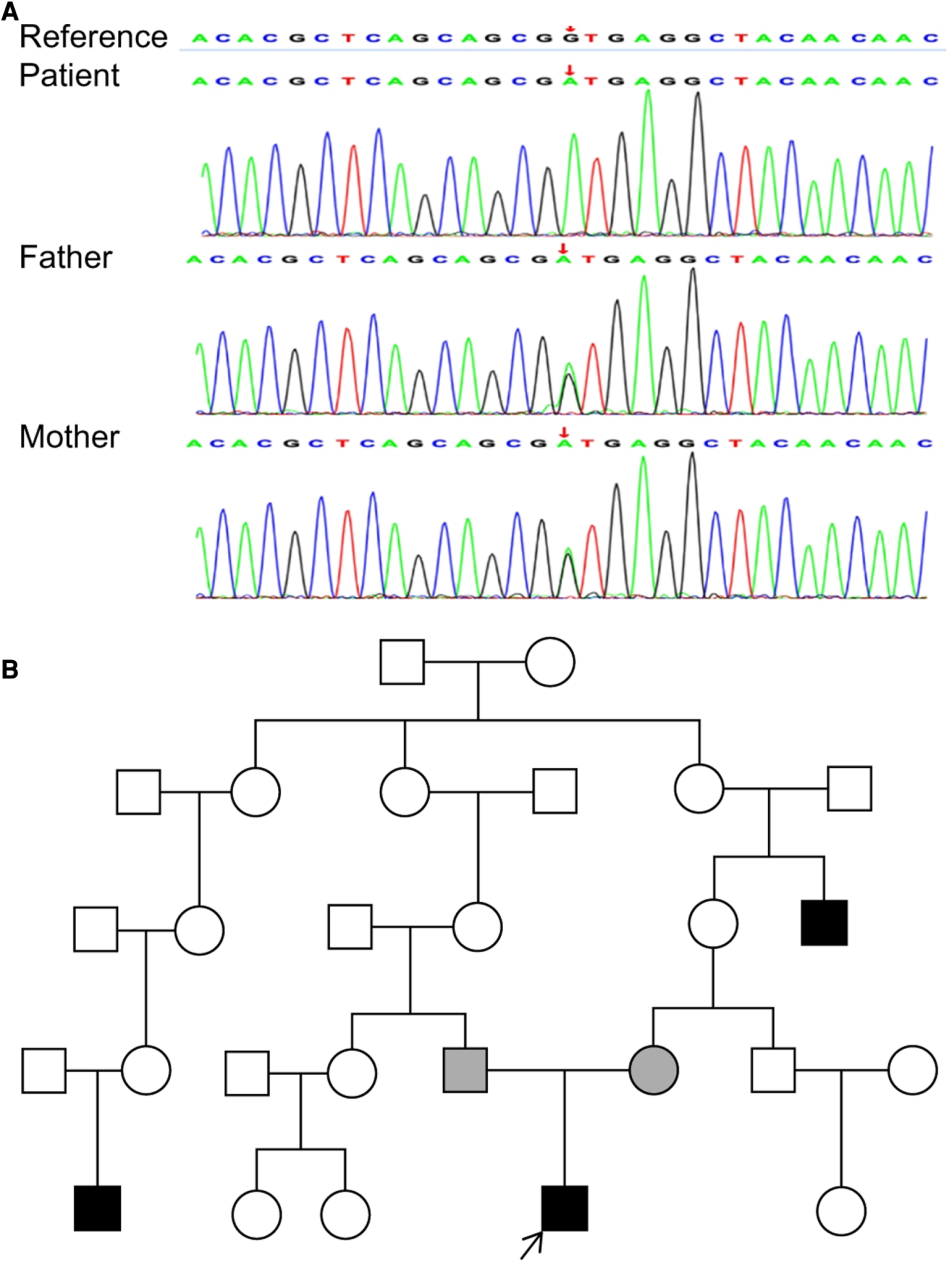
(**A**) sanger sequence confirmed NM_000860.5: c.498 + 1G > A in the patient and his parents. (**B**) The pedigree diagram of the affected family. Only the proband (arrow) and his parents underwent genetic tests.

We identified a homozygous HPGD(NM_000860.5):c.498 + 1G > A variant in the patient, and both parents were carriers of this variant (Figure 3A). The average sequencing depth and the average coverage of this variant were 121.60% and 100%, respectively. According to the ACMG clinical practice guidelines, the variant is pathogenic (PVS1 + PM2 + PP3). The LOF mutation leads to a possible loss of gene function (PVS1). The MAF of this variation is not more than 0.0005, which belongs to low frequency variation (PM2). Based on conserved and protein structure prediction software evidence, various statistical methods can be used to predict the effect of the mutation on the gene or gene product (GERP, phyloP20way, phastCons20way, MaxEntScan, splice_dbscSNV) (PP3). The variant has never been documented in published SNP databases or reported cases. In our case, one of the patient's cousins and granduncles had the same syndrome, but they did not undergo genetic testing because we could not contact them (Figure 3B).

## 3. Therapeutics assessment

The patient was diagnosed with PHO combined pulmonary lesions and intussusception. He was treated with naproxen orally to improve joint swelling and pain. The child was not treated for respiratory and gastrointestinal problems because of a lack of symptoms and other findings. In 2022, at age 11, his arthralgia was relieved when he revisited for further consultation. The patient complained of poor appetite, malnutrition, and no respiratory symptoms. Abdominal CT showed food retention in the stomach, duodenal dilation, lengthy and clustered intestines, and endoscopic biopsy suggested chronic inflammatory changes in the digestive tract (Figures 2G–M). Serum 25-hydroxyvitamin D [25(OH)D] and serum ferritin (SF) levels further decreased in the child since 2019, and peripheral blood leukocyte and hemoglobin levels also showed a decreasing trend (Table 1). The child was not treated for respiratory tract lesions as he remained free of respiratory symptoms, and imaging suggested no exacerbation of pulmonary lesions. The child, however, presented with evidence of exacerbated gastric and duodenal dilatation compared to previous observations in 2019. A literature review was conducted to assess the efficacy of various therapeutic options. However, there was a lack of clear evidence supporting the use of medication to address this symptom. No indication was found for emergent surgical intervention. Based on this analysis, it was recommended to administer a regimen of vitamins, calcium, and other nutritional supplementation to alleviate the child's symptoms of wasting and chronic malnutrition. The patient was advised to attend regular follow-up appointments to monitor his condition.

## 4. Discussion

Autosomal recessive PHO 1 (PHOAR1, MIM: #259100) is characterized by childhood-onset primary digital clubbing, osteoarthropathy, and acro-osteolysis, with variable features of pachydermia, delayed closure of the fontanels, and congenital heart disease due to biallelic *HPGD* mutations in some patients. Compared to those with PHOAR2 caused by *SLCO2A1* defects, patients with PHOAR1 are six times more likely to be males than females, with clinical features of early-onset hypertrophic osteoarthropathy, milder pachydermia, gastrointestinal symptoms, and lower prevalence of arterial catheters (5, 6). In this study, the patient's clinical findings met the diagnostic criteria for PHOAR1.

Previous studies have shown that gastrointestinal changes are common in patients with *SLCO2A1* defects, resulting in multiple gastrointestinal inflammations, such as watery diarrhea, Crohn-like lesions, etc (7–10). The patient presented with gastrointestinal dysfunction in the present study, including recurrent transient intussusception and nutritional malabsorption, which is putatively attributed to PGE2 levels. 15-PGDH inhibition (PGDHi) increases PGE2 levels, thereby modulating hematopoietic function. In mice, PGE2 regulates inflammation in the intestines; genetic ablation of 15-PGDH improves injury responses in the colon (11), while normal values for PEG2 levels help maintain the homeostasis of the gastrointestinal mucosa. In the present study, cross-sectional blood tests showed that the patient had continuously decreased levels of serum 25(OH)D and SF at the age of 7 and 10 years, with a downward trend of peripheral blood leukocytes and hemoglobin levels. Therefore, we speculated that the *HPGD* defect promotes gastrointestinal inflammation, malabsorption, active vitamin D decline, and blood cell reduction (Table 1). In addition, anemia and pancytopenia have been observed in some PHO cases (12).

Acro-osteolysis, a symptom of PHO, was also observed in our case. A reasonable theory is that chronically elevated levels of PGE2 due to *HPGD* deficiency lead to limb bone ablation by stimulating osteoclast activity on the one hand and bone resorption through the promotion of vascular endothelial growth factor (VEGF) (13).

There is currently no effective etiological treatment for PHOAR1. A previous study showed that combination therapy, including non-steroidal anti-inflammatory drugs, immunosuppressants, biological agents, and other treatment methods, could not improve the pestle fingers, joint enlargement, periosteum thickening, or skin thickening but might improve pain and inflammation, and surgery may improve facial skin enlargement and poor appearance caused by thickness (14). In most patients, the stabilization lasted 5–20 years (15). The patient in our case was treated with non-steroidal anti-inflammatory drugs and immunosuppressants for 2 years after his diagnosis until the joint pain was relieved, and his parents were concerned about the side effects of the drug. After 3 years, the patient's condition did not change.

Little practical experience in the treatment of PHO gastrointestinal lesions has been reported. Simplifying intestinal medication and prescribing factor diets may help improve patients’ intestinal function.Although he still had no gastrointestinal symptoms, the abdominal CT suggested dilatation of the stomach and duodenum that was not present before.Since no effective drug treatment has been identified and the drugs may also cause gastrointestinal side effects, after evaluating the benefits and risks of intestinal tract resection, the parents chose a conservative approach. The boy received a high-calorie and low-fiber diet and vitamins plus calcium supplements to reduce the enterogastric burden and improve nutrition. However, as the disease progresses, severe intussusception may develop, and an operation will be required. In addition, the patient received caffeic acid tablets orally to improve leukocytosis; however, the effect remains to be observed.

In summary, the etiology of PHOAR1 remains unclear, and the findings of vitamin D deficiency and acro-osteolysis in our case helped to understand the potential mechanism of the PGE2 pathway-mediated inflammation of the digestive tract in PHOAR1.

## Data Availability

The datasets presented in this study can be found in online repositories. The names of the repository/repositories and accession number(s) can be found in the article/Supplementary Material.
